# Major Risk Factors Analysis of Pruritus Complicated by Type 2 Diabetes Mellitus and the Effect of Comprehensive Nursing Intervention

**DOI:** 10.3389/fsurg.2022.842884

**Published:** 2022-02-15

**Authors:** Qiu Ping Yang, Yuan Yuan Chen, Zhenzhen Li, Mingming Xu

**Affiliations:** ^1^Endocrinology Ward, Shenzhen Traditional Chinese Medicine Hospital (The Fourth Clinical Medical School of Guangzhou University of Chinese Medicine), Shenzhen, China; ^2^Nursing Department, Shenzhen Traditional Chinese Medicine Hospital (The Fourth Clinical Medical School of Guangzhou University of Chinese Medicine), Shenzhen, China; ^3^Surgery Ward 3-4, Shenzhen Traditional Chinese Medicine Hospital (The Fourth Clinical Medical School of Guangzhou University of Chinese Medicine), Shenzhen, China

**Keywords:** type 2 diabetes mellitus, pruritus, risk factors, comprehensive care, pruritic medium

## Abstract

**Objective:**

To observe the main risk factors for pruritic skin evidence complicating type 2 diabetes mellitus (T2DM) and the effectiveness of interventions with comprehensive care measures.

**Methods:**

Two hundred and twenty four patients with T2DM admitted to our hospital from June 2020 to November 2021 were selected and divided into Diabetic pruritus group (DP group, *n* = 71) and T2DM group (*n* = 153) according to the patients' complications of pruritus. General information such as gender, age, body mass index (BMI), duration of illness, family history, treatment modalities, other comorbidities, underlying illnesses were collected from all patients. Fasting plasma glucose (FPG), renal function [Serum creatinine (Scr), urea nitrogen (BUN), uric acid (BUA)], lipid levels [total cholesterol (TC), triacylglycerol (TG), high-density lipoprotein cholesterol (HDL-C), low-density lipoprotein cholesterol (LDL-C)] were measured in all patients on day 2 after admission. Risk factors for pruritus complicating T2DM were identified by single multifactorial analysis. Meanwhile, patients in the DP group were divided into group A (*n* = 35) and group B (*n* = 36) using the random number table method. Group A adopted the conventional care mode and group B patients adopted the comprehensive care interventions to compare the care effects [visual analog score (VAS) before and after care, treatment efficiency, care satisfaction rate] of patients in groups A and B; the levels of pruritus mediator indicators [substance P,β-endorphin (β-EP) and γ-interferon (INF-γ)] before and after care.

**Results:**

Risk factors for pruritus in T2DM were age, duration of DM, combined Diabetic peripheral neuropathy (DPN), combined diabetic retinopathy (DR), combined diabetic kidney disease (DKD) and serum FPG levels (*P* <0.05). Satisfaction rate of nursing care, treatment efficiency, post-care improvement in VAS scores, serum substance P, β-EP and INF-γ levels and other mediators of pruritus were better in Group B with integrated nursing intervention than in group A with conventional care only (*P* < 0.05).

**Conclusion:**

Pruritus in T2DM is associated with age, duration of DM, combined DPN, combined DR, combined DKD and FPG levels. Comprehensive care according to the above risk factors can effectively relieve patients' clinical symptoms and signs, improve the level of pruritus mediators and patient-care relationship.

## Introduction

Diabetes mellitus (DM) is a group of metabolic disorders characterized by a chronic increase in plasma glucose levels due to a variety of causes (1, 2). Prolonged disturbances in the metabolism of carbohydrates, fats and proteins can be detrimental to multiple organ systems and lead to chronic progressive hypofunction of tissues and organs (3). The prevalence and incidence of DM have shown a dramatic increase in recently years, which is a worldwide public health problem that seriously threatens human health, according to the statistics of the International Diabetes Federation in 17 years (4). With the prolongation of the disease, long-term hyperglycemia can cause microvascular and neurological damage, resulting in a variety of complications. The skin is the organ with the most extensive distribution of nerves and blood vessels throughout the body, and the skin damage caused by diabetes is called diabetic skin disease (5).

Diabetic skin disease has a high incidence in DM patients, and diabetic pruritus is one of the common skin complications. Patients with DM complicated by pruritus usually show generalized or localized pruritus without primary skin damage, or secondary skin damage such as scratch marks, crusts, pigmentation and eczema-like changes after scratching (6, 7). Diabetic pruritus (DP) is often worse at night, which not only affects the quality of life and sleep, but also causes psychological and mental abnormalities such as irritability, anxiety and depression due to the unbearable itching symptoms (8, 9). Although pruritus in DM patients is not a direct threat to patients' lives, it has a serious impact on patient's quality of life and physical and mental health, reduces patients' compliance with treatment and is detrimental to the control of their condition, thus increasing their long-term mortality (10, 11). In recent years, there has been an increasing number of research treatments for pruritus in patients with MD, but with little success (12, 13). Therefore, analysis of the causes of pruritus in DM patients, establishment of a suitable assessment system, and interventions targeting them to evaluate the effects of interventions have great clinical and social significance in improving the quality of life of DP patients.

Based on such a background, this study examines both the causes of pruritus and interventions in patients with T2DM. On the one hand, the causes of pruritus in T2MD patients were analyzed through a survey of pruritus in T2MD patients in our hospital, while on the other hand, different modes of care were applied to patients with pruritus in T2MD who were treated in our hospital, thus helping to control the symptoms of pruritus in DP patients.

## Methods

### Subjects of Observation

Two hundred and twenty four patients with T2DM admitted to our hospital from June 2020 to November 2021 were selected and divided into Diabetic pruritus group (DP group, 71 patients) and T2DM group (153 patients) according to the patients' complications of pruritus. Inclusion criteria: 1. The diagnosis of T2DM was based on the 1999 WHO Expert Committee on Diabetes Mellitus diagnostic criteria (14). The diagnosis of DP (15) was based on dermatologic venereology: the patient has only pruritus without an obvious primary rash, which may be accompanied by dry skin, scratching and crusting, and the pruritus lasts for 2 weeks or more. Pruritus may be localized or generalized, including the trunk, lower legs, anus, and perineum. 2. had not applied corticosteroids within 2 weeks prior to admission and had not taken antihistamines or (and) topical corticosteroids within 1 week; 3. gave informed consent to the study care protocol. 4. Occupation for civil servants or retirees. Exclusion criteria: 1. pruritus caused by primary skin conditions such as psoriasis, eczema or atopic dermatitis; 2. patients who have used medication for pruritus or medication that can cause pruritus in the last 30 days; 3. patients with severe liver or renal abnormalities; 4. Patients with combined immune system diseases, Blood system diseases, neurological diseases and malignant tumors; 5. patients with combined severe psychological or psychiatric diseases. At the same time, patients in the DP group were divided into group A (35 cases) and group B (36 cases) using the random number table method, with group A taking conventional nursing measures during hospital treatment and group B taking comprehensive nursing interventions during hospital treatment.

### Research Methodology

General information on the patient's gender, age, body mass index (BMI), duration of illness, family history, treatment modalities, other comorbidities [Diabetic peripheral neuropathy (DPN), diabetic retinopathy (DR), diabetic kidney disease (DKD)], underlying illnesses, etc. were collected on the day of admission by means of a medical history enquiry. 10 mL of fasting peripheral venous blood was collected on day 2 after admission, and fasting plasma glucose (FPG), renal function [Serum creatinine (Scr), urea nitrogen (BUN), uric acid (BUA)] and lipid levels [total cholesterol (TC), triacylglycerol (TG), high-density lipoprotein cholesterol (HDL-C), low-density lipoprotein cholesterol (LDL-C)] were measured.

### Treatment Methods

Patients in Groups A and B received the usual symptomatic treatment for DP evidence on admission, including lowering blood glucose (insulin and oral hypoglycaemic drugs), improving vascular microcirculation (prostilbestrol injection), nerve nourishment (methylcobalamin injection), and anti-itch medication such as oven-glycerine lotion or (and) oral loratadine on the itchy skin.

### Nursing Care Methods

#### Group A

Routine nursing programme was adopted, i.e., keeping the room environment clean and quiet with appropriate room temperature and humidity to avoid irritation to the skin by various adverse factors; changing bed sheets and covers regularly to keep them dry and tidy; routine health education and psychological intervention.

#### Group B

Integrated intensive care interventions were used in Group A's, which included the following. ①Enhance skin care: The patients were instructed not to scratch the itchy areas repeatedly in order to maintain the integrity of the patient's skin and to clean the patient's skin regularly and the patient's family was instructed to relieve the patient's itching symptoms by patting and massaging, while communicating more with the patient to divert his attention. Distract the patient from the itchy skin by playing music, reading, exercising and playing recreational games to avoid or reduce the risk of aggravating the condition by scratching and rubbing, which can cause skin breakdown and infection. It is also important to keep the nails trimmed with curved edges to avoid breaking the skin when scratching. ②Strengthen psychological intervention: Patients with DP were mostly of advanced age and have a long disease duration, and they were prone to tension, anxiety, pessimism and other emotions when they were troubled by symptoms for a long time. Emotional de-escalation of the patient should be reinforced at this time, by communicating positively and enthusiastically with the patient, using empathy and breathing to reduce stress to distract the patient from the illness. Assist patients to develop a correct attitude toward the disease and maintain a good state of mind so that they can actively cooperate with treatment and care. ③Strengthen dietary regimen: High blood sugar can cause itchy skin in patients, so sugar control should be strengthened in the daily diet. Given the patient a high-protein, high-vitamin, low-fiber, easy-to-digest diet, maintain water-electrolyte balance, avoid spicy and stimulating foods and beef, sheep and seafood, and instructed the patient to quit smoking and limit alcohol. ④Strengthen medication care: standardize the medication according to the duration of action of different hypoglycaemic drugs, and monitor blood glucose regularly, pay attention to prevent adverse reactions such as hypoglycaemia and complications such as liver and kidney function impairment, allergy and oedema. If the blood sugar was not well controlled, contact the physician to adjust the treatment plan in a timely manner; if there was hyperalgesia and neuropathy, give the appropriate treatment and care according to the doctor's prescription; as the itching sensation is stronger at night, sleep-promoting drugs can be given. ⑤Strengthening health education: Through PPT lectures, seminars and health education booklets, we explain to the nursing clients the knowledge about pruritus complicated by DM, the serious adverse outcomes that may result from scratching behavior, the importance of active cooperation with treatment and the scientific diet of diabetes, so that the patients can grasp the relevant knowledge and develop health behaviors such as consciously controlling diet, taking medication on time and regularly monitoring blood sugar.

### Observation Indicators

#### Risk Factors

Information on gender, age, body mass index (BMI), disease duration, family history and treatment modality was collected from patients in the pruritus group and the T2DM group, and one-way and multi-way logistic regression analyses were used to explore the main risk factors for T2DM complicating pruritus evidence.

#### Effectiveness of Care

Information on pruritus scores, lesion healing time and treatment efficiency were collected and compared between the two groups before and after the nursing intervention. Pruritus scores were evaluated using visual analog scoring (VAS) (16). According to the severity of the patient's symptoms, a score from 0 to 10 was assigned from no itching symptoms to severe itching and inability to sleep, respectively, with higher scores indicating more severe itching symptoms. Satisfaction rates were measured using a hospital-made nursing satisfaction questionnaire and patients were surveyed after the nursing intervention The internal consistency Cronbach's α coefficient of the questionnaire was 0.823. The patients were classified into four levels, A, B, C and D, according to their satisfaction with the nursing interventions, representing “very satisfied,” “quite satisfied,” “average” and “dissatisfied,” respectively. The satisfaction rate is the percentage of the sum of the number of cases in each group of A, B and C grades compared with the total number of cases in each group. Treatment efficiency was assessed according to the relevant literature (17, 18). The efficacy of the patients was also divided into four grades, A, B, C, and D, which stand for “cure,” “significantly effective,” “effective” and “ineffective,” respectively, and the total effective rate is the percentage of the total number of cases in grades A, B, and C compared with the total number of cases in each group.

#### Pruritus Mediator Levels

5 mL of fasting peripheral venous blood was collected from patients before and after the nursing intervention, and the levels of pruritus mediators such as serum substance P, β-endorphin (β-EP) and γ-interferon (INF-γ) were determined by Enzyme-linked immunosorbent assay (ELISA) using a fully automated enzyme-labeled instrument and compared.

### Statistical Methods

The trial applied EXCEL to collate the relevant data, SPSS 20.0 was applied to calculate the statistical results of the data, and Prism 8.0 was applied to draw the pictures. The measurement data were expressed as mean ± standard deviation (±s), and if the data obeyed normal distribution, the paired *t*-test was applied to compare the difference between itself before and after treatment within the group, and the *t*-test of two independent samples was applied to compare the difference between treatment between groups; the count data were expressed as (*n*,%), and the χ^2^ test was used for non-rank count data, and the rank sum test was used for rank data. Logistic regression models were used to analyse the main risk factors for T2DM complicated by pruritus. *P* < 0.05 was taken as statistically significant.

## Results

### General Information of Patients in the DP Group Compared With the T2DM Group

A comparison of the information collected from DP patients and those with T2DM alone showed statistically significant differences in age, BMI, duration of DM, insulin use, combined hyperlipidaemia, combined DPN, combined DR, combined DKD, and serum levels of FPG, Scr, BUA and BUN (*P* < 0.05). There were no statistically significant differences between the two groups in terms of gender, combined hypertension, combined coronary artery disease, long-term smoking, alcohol abuse, serum levels of TC, TG, HDL-C and LDL-C (*P* > 0.05, see Table 1 for details).

**Table 1:** General information of patients in the DP group compared with the T2DM group [($\bar{x}$±s) (*n*,%)].

**Information**		**DP group (*n* = 71)**	**T2DMgroup (*n* = 153)**	***t/χ^2^*** **value**	***P-*** **value**
Gender	Male	42 (59.15)	87 (56.86)	0.104	0.747
	Female	29 (40.85)	66 (43.14)		
Age (years)		64.52 ± 12.18	58.77 ± 13.26	3.582	<0.001
BMI(kg/m^2^)		23.68 ± 5.79	21.81 ± 4.77	2.547	0.012
Duration of DM disease (years)		13.57 ± 7.64	10.09 ± 8.25	3.006	0.003
Family history of DM	Yes	30 (42.25)	49 (32.03)	2.222	0.136
	No	41 (57.75)	104 (67.97)		
Use of insulin	Yes	60 (84.51)	66 (43.14)	33.726	<0.001
	No	11 (15.49)	87 (56.86)		
Co-morbidities	Hyperlipidaemia	36 (50.70)	47 (30.72)	8.305	0.004
	Hypertension	48 (67.61)	94 (61.44)	0.795	0.373
	Coronary heart disease	14 (19.72)	31 (20.26)	0.009	0.925
Other complications	DPN	48 (67.61)	36 (23.53)	40.198	<0.001
	DR	43 (60.56)	34 (22.22)	31.602	<0.001
	DKD	45 (63.38)	32 (20.92)	38.767	<0.001
Chronic smoking	Yes	31 (43.66)	48 (31.37)	3.208	0.073
	No	40 (56.34)	105 (68.63)		
Alcohol abuse	Yes	26 (36.62)	41 (26.80)	2.232	0.135
	No	45 (63.38)	112 (73.20)		
FPG (mmol/L)		10.46 ± 2.58	7.09 ± 3.24	7.701	<0.001
Scr (μmol/L)		152.58 ± 96.42	80.22 ± 61.20	6.797	<0.001
BUA (μmol/L)		359.74 ± 113.25	303.82 ± 127.51	3.159	0.002
BUN (mmol/L)		10.58 ± 5.23	6.69 ± 3.42	6.642	<0.001
TC (mmol/L)		4.79 ± 2.06	5.17 ± 2.43	1.140	0.255
TG (mmol/L)		3.24 ± 1.52	3.48 ± 1.37	1.178	0.240
HDL-C (mmol/L)		1.14 ± 0.61	1.06 ± 0.70	0.828	0.409
LDL-C (mmol/L)		2.73 ± 1.09	2.90 ± 1.16	1.040	0.300

### Single Factor Logistic Regression Analysis of Pruritus in Combined T2DM

Age, BMI, duration of DM, use of insulin, comorbid hyperlipidaemia, comorbid DPN, comorbid DR, comorbid DKD, serum levels of FPG, Scr, BUA and BUN were used as independent variables (see Table 2 for assignments) and whether the patient had comorbid pruritus (assignments: 0 = no, 1 = yes) was used as the dependent variable in a one-way logistic regression analysis. The results showed that age, duration of DM, combined DPN, combined DR, combined DKD and FPG serum levels may be associated with combined pruritus in patients with T2DM (*P* < 0.05, Table 3).

**Table 2:** Assignment for multivariate analysis of factors.

**Influencing factors**	**Assignment**
Age	<65 years = 0, ≥65 years = 1
BMI	<24 kg/m^2^ = 0, ≥24kg/m^2^ = 1
Duration of DM	No = 0, Yes = 1
Using insulin	No = 0, Yes= 1
Combined hyperlipidaemia	No = 0, Yes = 1
Combined DPN	No = 0, Yes = 1
Combined DR	No = 0, Yes = 1
Combined DKD	No = 0, Yes = 1
FPG	<9 mmol/L = 0, ≥9 mmol/L = 1
Scr	<110 μmol/L = 0, ≥110 μmol/L = 1
BUA	<420 μmol/L = 0, ≥420 μmol/L = 1
BUN	<7.1 mmol/L=0, ≥7.1 mmol/L=1

**Table 3:** Single factor Logistic regression analysis of pruritus in combined T2DM.

**Influencing factors**	***B***	**SE**	**Walds**	**OR**	**95%CI**	***P-*** **value**
Age	0.082	0.09	92.245	1.085	1.066~1.105	<0.001
BMI	0.041	0.030	3.126	1.042	0.982~1.105	0.071
Duration of DM	0.517	0.159	9.453	1.677	1.228~2.290	0.002
Using insulin	0.364	0.251	2.015	1.439	0.880~2.354	0.153
Combined hyperlipidaemia	0.391	0.326	1.025	1.478	0.780~2.801	0.733
Combined DPN	1.022	0.231	26.38	2.779	1.767~4.370	<0.001
Combined DR	0.759	0.146	13.597	2.136	1.605~2.844	<0.001
Combined DKD	0.635	0.158	12.248	1.887	1.384~2.572	<0.001
FPG	0.543	0.102	10.258	1.721	1.409~2.102	0.001
Scr	0.151	0.213	0.230	1.630	0.766~1.766	0.611
BUA	0.058	0.051	0.633	1.060	0.959~1.171	0.510
BUN	0.129	0.323	0.124	1.138	0.604~2.143	0.836

### Multivariate Logistic Regression Analysis of Pruritus in Combined T2DM

The indicators with significant differences in the univariate analysis were used as independent variables (assigned as in the univariate analysis), and whether the patients had comorbid pruritus (assigned as in the univariate analysis) was used as the dependent variable in the multivariate logistic regression analysis, and the results showed that age, duration of DM, comorbid DPN, comorbid DR, comorbid DKD and serum FPG level were independent risk factors for combined pruritus in patients with T2DM (*P* < 0.05, Table 4).

**Table 4:** Multivariate logistic regression analysis of pruritus in combined T2DM.

**Influencing factors**	***B***	**SE**	**Walds**	**OR**	**95%CI**	***P-*** **value**
Age	0.074	0.011	40.875	1.077	1.054~1.100	<0.001
Duration of DM	0.504	0.139	10.146	1.655	1.261~2.173	0.001
Combined DPN	0.958	0.264	19.330	2.606	1.554~4.373	<0.001
Combined DR	0.744	0.128	13.112	2.104	1.637~2.704	<0.001
Combined DKD	0.336	0.106	5.879	1.399	1.137~1.722	0.011
FPG	0.487	0.125	7.326	1.627	1.274~2.079	0.009

### Comparison of Care Outcomes Between Group A and Group B

**Figure 1:**
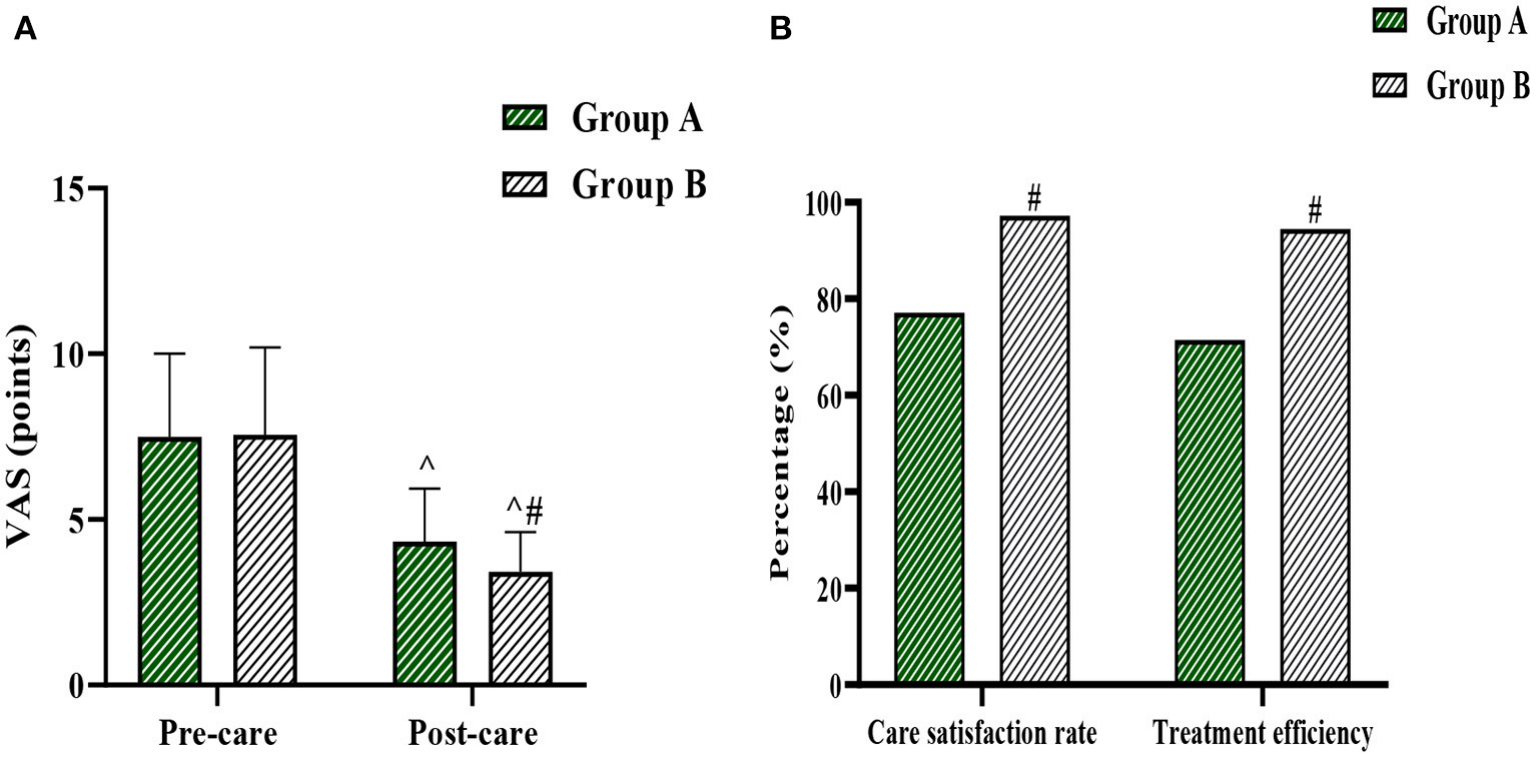
**(A)** shows the comparison of VAS scores between the two groups, and **(B)** shows the comparison of nursing satisfaction rate and treatment efficiency between the two groups. Compared with the same group before care, ^*P* < 0.05, and compared with the same period in Group A, #*P* < 0.05.

The pre-intervention VAS score, post-intervention VAS score, nursing satisfaction rate, and treatment effectiveness rate in group A were (7.49±2.51) points, (4.33 ± 1.60) points, 77.14, and 71.43%, respectively. The pre-intervention VAS score, post-intervention VAS score, nursing satisfaction rate, and treatment effectiveness rate in group B were (7.56 ± 2.63) points, (3.42 ± 1.20) points, 97.22, and 94.44%, respectively. The difference in VAS scores between the two groups before the intervention was not statistically significant (*P* > 0.05), and the VAS scores after the intervention were significantly lower than those before the intervention in both groups (*P* < 0.05). The VAS scores after the intervention in group B were lower than those in group A, and the satisfaction rate of care and treatment efficiency were higher than those in group A, and the difference was statistically significant (*P* < 0.05) (Figures 1A,B).

### Comparison of Serum Pruritic Mediator Levels Between Group A and Group B

**Figure 2:**
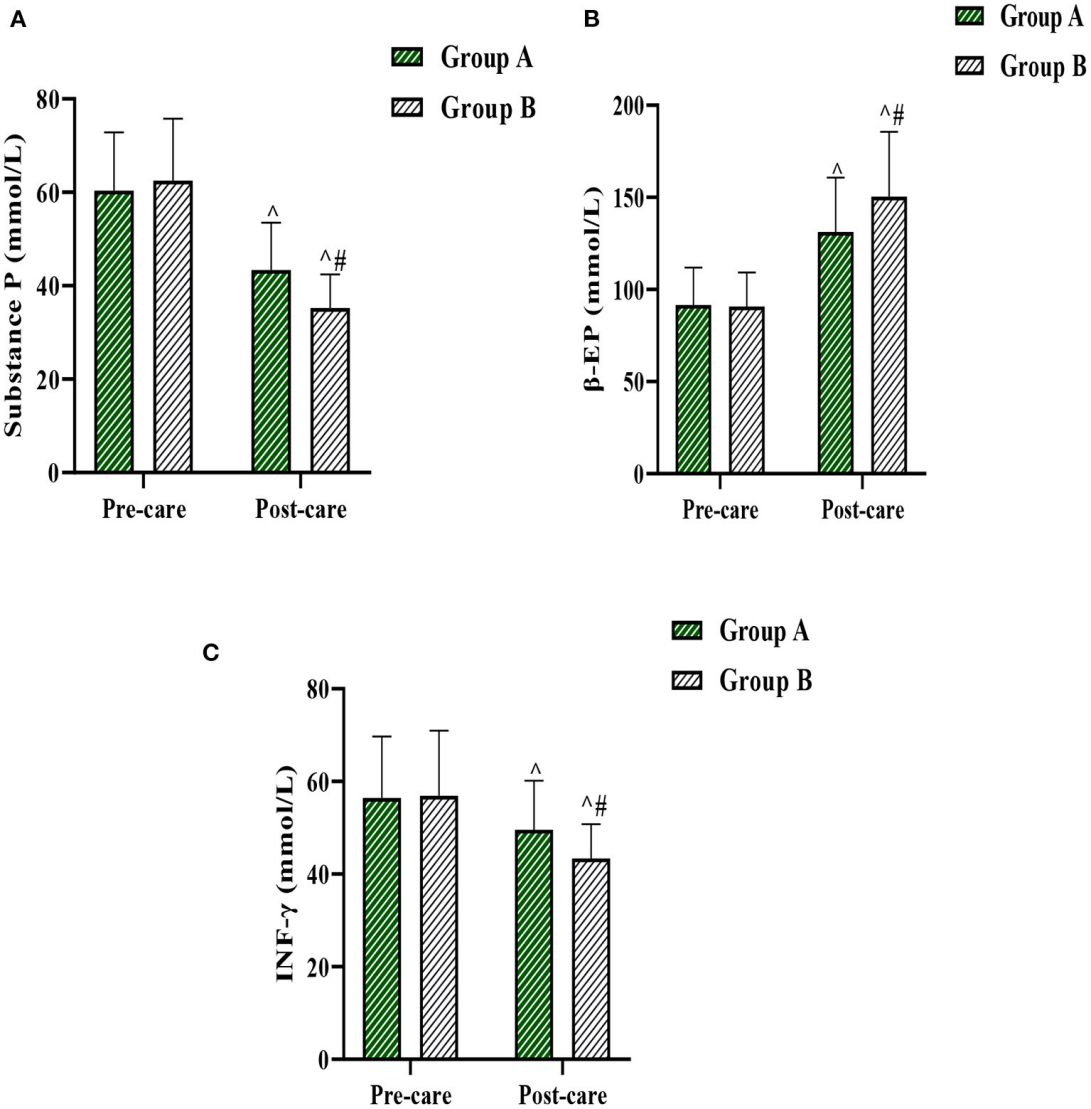
**(A–C)** represent the comparison of substance P, β-EP and INF-γ levels before and after care in the two groups, respectively. Compared with the same group before care, ^*P* < 0.05, and compared with the same period in Group A, #*P* < 0.05.

The serum pruritic mediators levels such as substance P, β-EP and INF-γ in the two groups before and after the intervention were compared. The results were as follows: There was no statistical difference in the levels of serum substance P, β-EP, and INF-γ between the two groups before the nursing intervention. Academic significance (*P* > 0.05). After the nursing intervention, the levels of serum substance P and INF-γ in both groups decreased significantly, and the levels of serum β-EP increased significantly in both groups (*P* < 0.05); the levels of serum substance P and INF-γ in group B after the nursing intervention were significantly lower than those in group A, and the levels of serum β-EP were significantly higher than those in group B (*P* < 0.05) (Figures 2A–C).

## Discussion

With rising living standards and an aging population, the prevalence of diabetes is increasing worldwide, with serious implications for health systems worldwide. Increased blood glucose causes damage to many types of cells such as endothelial cells, neurons, renal cells, fibroblasts and keratin-forming cells, leading to multisystem damage and various complications, of which pruritus is one of the common complications of DM, accounting for approximately one third of the incidence (19–21). The main clinical manifestations of DP are pruritus, or with secondary scratching, crusting, hyperpigmentation, secondary eczema-like and mossy lesions. Although these symptoms do not endanger patients' life and health, patients are prone to negative emotions such as anxiety and pessimism, and even rebellious psychology and do not actively cooperate with treatment, which eventually leads to the prolongation and aggravation of the disease. Therefore, it is necessary to improve the behavior and psychology of patients with DP with active and appropriate nursing interventions in the clinical treatment (22, 23). The previous model of care, which was based on symptomatic treatment and compliance with medical advice, lacked specificity and was not effective in the further development of the patient's pruritus condition (24). Moreover, the observation of risk factors for DM complicated by pruritus is still rare in the domestic and international literature, so this study aimed to identify the main risk factors for DM patients complicated by pruritus, in order to provide theoretical guidance for identifying corresponding nursing countermeasures. In addition, T2DM is about 90% of DM patients in China (25), thus the present research was performed focusing on T2DM patients.

The results of this study showed that T2DM patients with high age, high BMI, long duration of DM, insulin use, combined hyperlipidaemia, combined DPN, combined DR, combined DKD, high serum FPG, Scr, BUA and BUN levels had a higher prevalence of pruritus. Further results from a single multifactorial analysis showed that age, duration of DM, combined DPN, combined DR, combined DKD and FPG levels were the main risk factors for pruritus in patients with T2DM. The higher risk of pruritus in elderly T2DM patients may be related to factors such as aging, atrophy and thinning, dryness, reduced function of various glands, poor repair capacity, reduced skin barrier function and susceptibility to external irritation in the elderly population (26). With the prolongation of diabetes, the islet function of T2DM patients gradually declines, making it more difficult to control blood glucose, while poor blood glucose control can further reduce islet function to form a vicious circle (27). When the patient is in hyperglycemic state, the body tissues and plasma are in a hypertonic state, water flows autonomously and diffuses into the tissue fluid or plasma, the skin surface cells undergo dehydration effect, which stimulates the vegetative nerves and nerve endings, thus causing pruritus, and the degree of dry skin and pruritus increases with the increase of water loss (28, 29). Diabetic complications such as DPN, DR and DKD can involve the central nervous system, peripheral nerves and autonomic nerves, causing malfunction of sweat gland secretion. This is manifested by abnormal skin perspiration, loss of nourishment of the stratum corneum and dry and damaged skin, which can cause pruritus once it is attacked by microorganisms and external stimuli (30–32). In addition, study (33) showed that the longer the duration of DM, the higher the probability of patients developing complications such as DR, DKD and DPN, further suggesting that the duration of DM is a risk factor for pruritus.

Meanwhile, this study observed the effectiveness of comprehensive care in patients with T2DM complicated by pruritus, and the results showed that satisfaction rate of nursing care, treatment efficiency, post-care improvement in VAS scores, serum substance P, β-EP and INF-γ levels and other mediators of pruritus were better in Group B with integrated nursing intervention than in group A with conventional care only. It is suggested that comprehensive care during the treatment of patients with DP could improve their pruritus symptoms more effectively, reduce the level of serum pruritus mediators, ensure the treatment effect and increase the satisfaction rate of the patients with the care work.

In conclusion, pruritus in patients with T2DM is closely related to age, duration of DM, combined DPN, combined DR, combined DKD and FPG levels, and comprehensive care based on these risk factors can effectively alleviate patients' clinical symptoms and signs, improve the level of pruritus mediators and nurse-patient relationship. However, there are many limitations in this study, such as small sample size and lack of attention to long-term effects, which need to be further explored in the future with a larger sample size and more time, in order to provide a strong reference for the clinical care of patients with DP.

## Data Availability Statement

The original contributions presented in the study are included in the article/supplementary material, further inquiries can be directed to the corresponding author.

## Ethics Statement

The studies involving human participants were reviewed and approved by the Ethical Committee of Shenzhen Traditional Chinese Medicine Hospital (The Fourth Clinical Medical School of Guangzhou University of Chinese Medicine). The patients/participants provided their written informed consent to participate in this study.

## Author Contributions

All authors have made equal contributions to this study. QY is responsible for the writing of the paper. YC is responsible for the design of the study. ZL is responsible for the collection of cases and the implementation of the study. MX is responsible for the guidance of the whole study. All authors contributed to the article and approved the submitted version.

## Conflict of Interest

The authors declare that the research was conducted in the absence of any commercial or financial relationships that could be construed as a potential conflict of interest.

## Publisher's Note

All claims expressed in this article are solely those of the authors and do not necessarily represent those of their affiliated organizations, or those of the publisher, the editors and the reviewers. Any product that may be evaluated in this article, or claim that may be made by its manufacturer, is not guaranteed or endorsed by the publisher.
